# Workplace‐Based Education Interventions for Managing Metabolic Syndrome in Low‐ and Middle‐Income Countries: A Realist Review

**DOI:** 10.1002/puh2.224

**Published:** 2024-07-23

**Authors:** Sitotaw Kerie Bogale, Haribondhu Sarma, Tilahun Tewabe Alamnia, Matthew Kelly

**Affiliations:** ^1^ The National Centre for Epidemiology & Population Health The Australian National University Canberra Australian Capital Territory Australia; ^2^ College of Medicine and Health Sciences Bahir Dar University Bahir Dar Ethiopia

**Keywords:** contextual factors, education interventions, lifestyle, low‐ and middle‐income countries, metabolic syndrome, realist review, workplace health promotion

## Abstract

**Background:**

Sedentary office work and work‐related stress increase the risk of metabolic syndrome. Workplace‐based education interventions for promoting prevention are gaining popularity due to their positive impact on managing metabolic syndrome. We conducted this realist review to understand the contextual factors and mechanisms that contribute to the effectiveness of these interventions and how they interact to produce outcomes.

**Method:**

We conducted a comprehensive search of five main databases (PubMed, Web of Science, ProQuest, Scopus and PsycINFO) and Google Scholar, as well as references of included articles. We included all studies published until 12 January 2023, reporting the effects of workplace lifestyle education interventions on metabolic syndrome. Using a realist review approach, we identified and evaluated middle‐range theories to develop a context–mechanism–outcome configuration.

**Results:**

We identified 6883 titles for screening, of which 15 studies were included in this realist review. This realist review has identified strong social support networks, workplace influence, involvement of worksite managers and cultural relevance as contextual factors that contribute to the effectiveness of workplace‐based education interventions for managing metabolic syndrome in low‐ and middle‐income countries, which may not be as prominent in developed countries.

**Conclusion:**

The review concludes that while developing and implementing healthy lifestyle policies in the workplace, policymakers and researchers should consider social support, workplace influences, manager participation and cultural relevance.

## Graphical abstract

Strong social support networks, supportive workplaces, involvement of worksite managers and cultural relevance contribute to the effectiveness of workplace‐based education interventions for managing metabolic syndrome in low‐ and middle‐income countries.

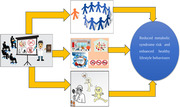

## 1 Background

Low‐ and middle‐income countries (LMICs) are experiencing significant impacts from non‐communicable diseases (NCDs); each year, more than three‐quarters of global NCD deaths, or 31.4 million, occur in LMICs [1]. Cardiovascular diseases and diabetes account for a significant portion of this NCD burden [2]. Cardiovascular diseases account for 44%, and diabetes accounts for 5% of all NCD deaths [3]. The risk of these two diseases is increased by metabolic syndrome, characterized as a group of conditions that includes obesity, insulin resistance, glucose intolerance, impaired regulation of body fat and high blood pressure (BP) [4]. Diabetes, cardiovascular disease and metabolic syndrome share a collection of risk factors, including physical inactivity, unhealthy diet, tobacco use and alcohol consumption [1, 5].

Metabolic syndrome is estimated to affect over a billion people worldwide [6]. In LMICs, metabolic syndrome prevalence is increasing rapidly as a result of growing urbanization, which drives changes in diets and physical activity [7]. A key aspect of changing lifestyles in LMICs is changing workplaces. Sedentary office work is becoming more common, and this, along with workplace‐related stress, is associated with a high risk of metabolic syndrome [8]. This makes the office workplace an ideal setting to implement healthy lifestyle education interventions on physical activity, healthy diet, stress management and avoidance of harmful alcohol consumption and smoking to prevent the development of metabolic syndrome and cardiovascular risk factors [9, 10, 11]. The workplace is a crucial setting for putting lifestyle education interventions into practice and offers special benefits [12, 13], including increased participant engagement, peer support and cost‐effectiveness [14, 15, 16], as well as access to a consistent and specific demographic group in a managed environment that spends a lot of time there [12, 17–20]. Workplace health promotion (WHP) initiatives therefore have the potential to make a significant impact on lowering the prevalence of NCDs and related risk factors [21].

WHP is defined as ‘the combined efforts of employers, employees, and society to improve the health and well‐being of people at work’ [22] and refers to initiatives involving primary prevention best practices, such as developing a healthy diet and increasing physical activity [23]. The Centres for Disease Control and Prevention (CDC) has also promoted the Workplace Health Promotion Program (WHPP) as a planned, organized and comprehensive set of programmes, policies, benefits and environmental supports based on workers’ needs [24].

Due to their advantages for health and work outcomes, interventions about preventative promotion in the workplace, involving activities targeting lifestyle choices, nutrition and/or physical activity, as well as overall well‐being, are expanding and becoming more popular [23]. These WHP interventions effectively manage metabolic syndrome, but the effectiveness is influenced by different contextual factors, mechanisms and their interactions. There is a lack of comprehensive evidence on these contextual factors and mechanisms, as well as their interactions, especially in LMICs. Thus, the objective of this review was to provide comprehensive evidence on contextual factors, mechanisms and how they interact to contribute to the effectiveness of workplace‐based lifestyle education interventions for managing metabolic syndrome among employed adults in LMICs.

## 2 Methods

### 2.1 Realist Method and Initial Search

Realist reviews use a theory‐driven methodology and a generative conceptualization of causality, concentrating on the development of middle‐range theories about context–mechanism–outcome (CMO) configurations [25]. These CMO theories explain how mechanisms operate in specific contexts to produce particular outcomes, clarifying why an intervention may be successful in one situation but not another [26, 27]. However, the relationships among CMO configurations do not guarantee fixed outcomes, as they are influenced by the decisions and interactions of people impacted by the intervention and its environment [28, 29, 30].

### 2.2 Literature Search

An initial scoping review identified potential theories and a general overview of the literature on lifestyle education interventions aimed at managing metabolic syndrome and its components among employed adults in LMICs. To find pertinent empirical studies, we conducted the main search to systematically identify studies on workplace‐based lifestyle education interventions to manage metabolic syndrome and its components among employed adults in LMICs by established search strategies, using different combinations of terms related to metabolic syndrome, employed and education. Five databases (PubMed, Web of Science, ProQuest, Scopus and PsycINFO) and 85 search terms were used between 01 December 2022 and 12 January 2023, and no time limits were applied to the resulting articles (Supporting Information File 1). Titles and abstracts were screened on the basis of the inclusion and exclusion criteria described in Table 1. Google Scholar, reference lists of screened studies, grey literature and thesis databases were also manually searched to identify additional pertinent original research studies.

**TABLE 1:** Inclusion and exclusion criteria for identifying empirical studies.

**Inclusion criteria**	**Participants:** Employed adults (≥18 years old) with metabolic syndrome or its components **Setting:** Low‐ and middle‐income countries refer to countries with a gross national income per capita between US$1135 or less and US$13,845, according to the World Bank in 2022 **Study design:** Empirical research evaluating the effectiveness of the intervention (randomized control trials [RCTs], cluster RCTs, quasi‐experimental studies, pre–post‐intervention longitudinal studies [with or without comparison groups]) **Types of data:** Both quantitative and qualitative, both published and grey literature (such as websites, reports of international organizations as well as dissertations and thesis) **Outcomes:** Primary (impact on metabolic syndrome and its components and healthy lifestyle behaviours), secondary and process outcomes **Year of publication:** No time limits for the year of publications
**Exclusion criteria**	Studies set in high‐income countries Protocols or reports of intervention development with no published evidence outcome Studies not published in the English language Feasibility and pilot studies with no outcome for the intervention

Titles, keywords and abstracts were screened by the corresponding author (SKB) to detect relevant studies using the established inclusion criteria shown in Table 1. A second reviewer (TTA) also autonomously screened retrieved studies. Studies were kept for full‐text screening if the reviewers could not agree. Full texts of 201 studies were retrieved and individually screened by SKB and TTA. Any discrepancies between SKB and TTA regarding the eligibility of studies were settled through further discussion.

### 2.3 Data Extraction From Empirical Studies

We extracted data (Supporting Information File 2) from the included studies on the following topics: research design, participant sample, setting, outcome measures; intervention description, components and study duration; intervention effect; authors’ programme theory where stated; and our interpretation of whether the study worked as intended.

Where authors did not explain how they believed their intervention would operate, we deduced this information by carefully reviewing the intervention's description. We also referred to associated study protocols or supporting documents such as trial process evaluations where applicable. SKB and TTA individually extracted data before comparing the data to iron out any discrepancies. The risk of bias was also assessed by SKB and TTA independently using the JBI checklists [31], which was created for the methodological quality assessment of randomized control trial studies and quasi‐experimental research that incorporates the criteria of inclusion (Supporting Information Files 3 and 4). Any discrepancies between the two reviewers’ assessments of the studies were worked out through discussion.

### 2.4 Developing a Theoretical Model to Understand How an Intervention Works

Relevant theories and models, primarily from health behaviour and psychology [32], were identified, and connecting intervention components to well‐established theories to develop the final CMO configurations (CMOcs) was also involved. This helped explain how and why the intervention designs contributed to effectiveness (Table 2). The identified theories were also evaluated using a higher level model [26, 33].

**TABLE 2:** Identified theories and information extracted from the literature used us to arrive at our final context–mechanism–outcomes configuration.

Design of education intervention	Established theory
**Seminar and reading materials** [14, 43, 45]**:** Aid the participants in acquiring knowledge, and skills, and promoting a healthy lifestyle **Sharing printed information** [41, 52]**:** Assisted them in improving knowledge, attitude, subjective norm, self‐efficacy, intention and practice **Self‐monitoring** [43, 48]**:** This helped them set realistic goals, track progress and evaluate their strengths and weaknesses, motivating them by enhancing self‐awareness **Feedback** [43, 45–47]**:** Make the participants motivated and adhere to the intervention	**The rational model** [53]**:** Whereby an individual's behaviour will change as their knowledge increases **Theory of planned behaviour** [54]**:** Whereby intention, norms, attitudes and self‐efficacy can influence individual behaviour **Self‐regulation theory** [55]**:** Behaviour change happens when people guide their thoughts, behaviours and feelings to achieve goals **Goal‐setting theory** [56]**:** The task difficulty is moderated by participants’ pursuit of a specific goal **Brouwer et al**. [57] – ‘Offering iterative tailored feedback has the potential to improve adherence’
**Reminders** [38, 41, 42, 44, 46, 47, 49]**:** Boosted intervention adherence, encouraged activity, advocated for a healthy diet and fostered social connections supporting confidence, security, and motivation to change	**Theory of interpersonal relationships** [58]**:** People's interactions with others, especially significant others, determine their sense of security, self, and the dynamics that motivate their behaviour **Brouwer et al**. [57] – ‘Participants will be more likely to stick with the intervention if they receive email reminders’
**Health screening** [14]**:** These alerts motivated action as cues	**Health belief model** [59]**:** Factors like perceived vulnerability, severity, benefits, barriers, cues, and self‐efficacy impact health behaviour
**Group activities** [45]**:** Fostered engagement and improved participants’ ability to identify a healthy diet	**Motivational learning theory** [60]**:** Intrinsic and self‐determined extrinsic motivation are sustained by satisfying the psychological needs of autonomy, competence and relatedness
**Workplace tobacco control regulations** [37, 47]**:** Enforced smoke‐free areas and promoted quitting	**Socioecological models** [61]**:** Human behaviour is influenced by repetitive patterns of activity in structured environments
**One‐to‐one counselling** [40]**:** Addresses the specific needs of each client	**Proper et al**. [62]**:** Workplace counselling improves health outcomes
**Participant characteristics and exposure to the intervention:** Drive them to engage in healthy behaviours	**Collado‐Mateo et al**. [63] ‘One of the key factors to increase adherence to physical exercise is initial exploration of participant's characteristics’
**Wide range of contents:** Enables them to access detailed information **Long duration:** Enables them to have sufficient time for behaviour modification **Expert to conduct the intervention:** Helps develop healthy lifestyle knowledge and skills **Multilevel approach:** Enables them to have social support	**Collado‐Mateo et al**. [63] – ‘Adherence to exercise is influenced by program characteristics, multidisciplinary involvement, participant education, realistic expectations and social support’
**A culture that discourages smoking:** Encourages intervention adherence and quitting smoking	**Nichter** [64] – ‘Changing cultural norms can help fight tobacco use’

The capability, opportunity and motivation for behaviour change (COM‐B) model, a higher level framework for behaviour change, was used to identify the necessary changes for the behaviour change intervention to be effective [34, 35].

COM‐B model identifies three necessary conditions for any behaviour to occur: capability, which includes physical skills, knowledge, behavioural regulation and memory, attention and decision‐making; opportunity, which includes environmental context, resources and social influences; and motivation, which includes beliefs about consequences, optimism and capabilities as well as reinforcement and emotion [36]. We used this higher level model to evaluate those identified theories, which provides insights into their potential usefulness and applicability in a given context (Table 3).

**TABLE 3:** Mechanisms of change capability, opportunity and motivation for behaviour (COM‐B mechanisms) evident in included empirical studies.

Study author	Intervention components used in each study	Capability	Opportunity	Motivation
Jalal et al. [42]	‐ Information ‐ Brochures ‐ Modules ‐ Assignments ‐ SMS messages	Knowledge and skill on healthy cooking and exercise techniques (behavioural capability) gained from information about healthy nutrition and physical activity skills. Assignments taught them how to apply the techniques in their daily lives. SMS messages improve memories	Resources used to teach and help people remember the critical elements of interventions include modules, assignments, pamphlets and SMS messages	Modules were seen as incentives, and they were taught to reward themselves by making time to relax (reinforcement). The assignments combined instructional content with behaviour modification strategies (which included using positive reinforcement and behaviour splitting, to help increase self‐efficacy)
Jamal et al. [43]	‐ Seminars, handouts ‐ Submission of records and log for each session ‐ Self‐monitoring activities ‐ Feedback	Knowledge, memory and skill on healthy behaviours obtained from seminars and reading materials	The availability of reading materials, for example handouts	Feedback encouraged them to stick with the intervention. The submission of diet records, a physical activity log and self‐monitoring activities motivated them to act
Shrivastava et al. [44]	‐ Sessions and training ‐ Reinforcement ‐ Need‐based advice ‐ Providing pedometers ‐ Text messages ‐ E‐mails ‐ Repeated phone calls	Sessions and training increase the participants’ knowledge, attitude and practices to get the expected results	The availability of pedometers to support physical exercise. Text messages, emails and repeated phone calls create the chance to establish an interpersonal relationship	Reinforcement and need‐based advice encourage to adhere the recommended intervention Interpersonal relationships fostered by text messages, repeated phone calls and emails motivate them to improve their behaviour
Eng et al. [14]	‐ Educational seminars and health exhibitions ‐ Lifestyle counselling ‐ Referral for medical treatment ‐ Followed up with subsequent counselling ‐ Providing brochures	Seminars, health exhibitions and brochures about health help people learn more	When necessary, the participants have the opportunity to receive medical treatment	Face‐to‐face lifestyle counselling and continued counselling after the session encourage people to act
Ramli et al. [45]	‐ Group exercise ‐ Workout sheet ‐ Group seminars ‐ Feedback ‐ Interactive activities, including quiz	Gains in knowledge and skills from seminars, group exercises and feedback	Social support from group seminars and exercise	They become motivated and adhere when given feedback and are given engaging tasks
Jaime et al. [46]	‐ Practical recommendation ‐ Reminder emails ‐ Feedback	Information from recommendations and emails asking recipients to report their self‐measured weights impact behaviour regulation	Presence of reminders and software to report their self‐measured weights	Emails reminding recipients to report their weights inspire action, and feedback encourages participation in suggested interventions
Anthony et al. [37]	‐ Reading materials ‐ Organized work–break exercises ‐ Sports competitions ‐ Mountain‐climbing events ‐ Distributed vegetable seeds and fertilizer and training on vegetable cultivation ‐ Created marked walking paths around worksites ‐ Smoking bans in the workplace ‐ Incentives	Gaining knowledge from posters and smoking prohibitions at work encourages them to control their behaviour	Opportunities are established by the availability of planned workplace exercises, the distribution of vegetable seeds and fertilizer and the creation of designated walking paths surrounding work locations	Sports competitions, mountain‐climbing events and incentives motivate the participant
Wang et al. [47]	‐ lectures, posters and text messages ‐ Nutrition education ‐ Tobacco control regulation in the workplaces ‐ Modifying workstations and office layouts, accessible indoor or outdoor sports facilities ‐ Feedback	Lectures, posters, text messages and informational materials about healthy eating and nutrition boost employees’ knowledge and skills, and workplace policies against smoking encourage them to regulate their behaviour	Tobacco control regulation in the workplaces and accessible indoor or outdoor sports facilities, including an indoor walking path, gives opportunities to adhere to healthy behaviour	Changes to workstations and office designs, yearly health exams to identify significant risk factors and feedback encourage the enactment of behaviour change
Nooritajer [38]	‐ Face‐to‐face interview on essential guidelines ‐ Booklet presentation ‐ A telephone call to them biweekly	The booklet presentation gives them information about the nutrition style	The participants have the option to call the researcher and ask any Questions they may have about the educational booklet	A face‐to‐face interview may inspire them to maintain a healthy nutrition style
Ribeiro et al. [48]	‐ General advice ‐ Counselling ‐ Booklet ‐ Dairy ‐ Pedometer	Advice, counselling and booklets offer information on how to boost PA in daily life and assist in bringing about behavioural change	They have the opportunity to self‐monitor their physical activity with pedometers	Having a diary to record their total daily steps encourages them to act
Soon et al. [49]	‐ lectures and group discussion ‐ A telephone call reminder ‐ A diary ‐ Booklet	They learn about the health advantages of physical activity, tactics and recommendations to improve physical activity, advantages of walking and techniques to raise the number of daily steps through lectures, group discussions and booklets	Participants have reminder telephone calls to do the suggested actions	Participants may be more motivated to act if they are required to bring their step diaries to each lecture and discussion session for assessment and feedback
Boshtam et al. [39]	‐ Education ‐ Modification of food menus and ingredients ‐ Planning on physical exercise during working hours ‐ Prohibition of smoking as a rule	Knowledge of healthy eating and exercise is improved through education using books, CDs and posters	Allow opportunity to lead a healthy lifestyle is increased by changing food menus and ingredients, engaging in exercise during working hours and generally forbidding smoking	Planning to incorporate physical activity and exercise during working hours encourages participation
Zahtamal et al. [50]	‐ Policy briefing, skills training ‐ Discussion and lectures ‐ Personal‐counselling ‐ Demonstration ‐ Reinforcement ‐ Information	Provide more information and improve knowledge, skill, attitude, subjective norm, self‐efficacy, intention and practice	Create chances for organizational and social support	Have reinforcement to keep them motivated
Wu et al. [40]	‐ One‐on‐one dietary guidance	Have information about diet	Opportunity to receive guidance based on needs	Need‐based guidance encourages adherence
Sinaga et al. [41]	‐ PowerPoint presentation ‐ Facilitated group discussion ‐ Brochure ‐ Reminder text messages	Reminder messages aid in memorization	Chance to see a real case as an example	Observing real cases motivates them to adhere to the intervention

This review is conducted following the realist and meta‐narrative evidence syntheses (RAMESES) reporting guidelines [28] (Supporting Information File 5).

## 3 Results

### 3.1 Search Results

**FIGURE 1:**
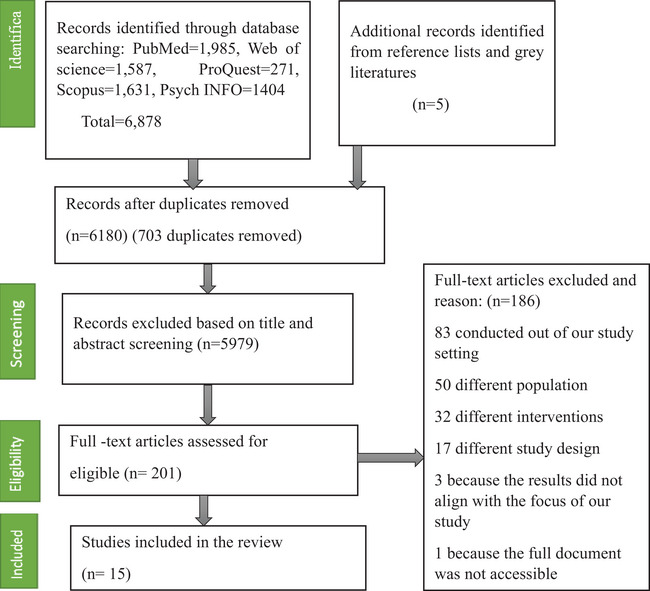
Study selection flow diagram of workplace‐based education interventions for managing metabolic syndrome in low‐ and middle‐income countries for systematic reviews in 2022.

A total of 6883 studies were identified through database and manual searches, including the grey literature and reference lists of included studies. Then duplicate articles (*n* = 703) and ineligible studies (*n* = 5979) were excluded on the basis of the titles and abstracts. Two hundred and one studies were included for the full‐text review and screening. Of these articles, 83 were removed because the studies were conducted outside of our study setting, 50 because they contained different populations, 30 because of the different intervention, 17 because of different study designs, 3 because the results did not align with the focus of our study and 1 because the full document was not accessible. Finally, 15 studies were included in this realist review. The study selection diagram is indicated in Figure 1.

### 3.2 Characteristics of the Studies

Out of the 15 articles that were included, 4 addressed studies in Malaysia, 3 in Iran and 2 in Brazil. The rest were carried out in Taiwan, China, India, Indonesia, Mexico and Ethiopia. Ten of the 15 included studies were randomized control trials and 5 were quasi‐experimental.

Six studies measured only primary outcomes, such as BP [14], reduced tobacco use, increased physical activity, improved dietary intake [37], nutrition style, body mass index (BMI) [38], BP, fasting glucose (FG), high‐density lipoprotein‐cholesterol (HDL‐C), low‐density lipoprotein‐cholesterol (LDL), obesity, waist circumference, total cholesterol [39], BMI, waist circumference, body fat, BP, total cholesterol, HDL‐C, triglycerides (TG) [40], BP, FG, HDL, LDL, obesity, waist circumference and total cholesterol [41]; nine studies measured their secondary outcomes in addition to primary outcomes [42, 43, 44, 45, 46, 47, 48, 49, 50]; five studies reported process outcomes [42, 43, 47–49, 51]. The process outcome that was most frequently reported was adherence to the intervention. Only five studies reported all primary, secondary and process outcomes [42, 43, 47–49] (see Supporting Information File 2 for detailed information).

Eleven studies provided reading materials (booklets, pamphlets, leaflets and posters) in addition to face‐to‐face education and/or counselling on healthy lifestyles [14, 37–39, 41–43, 47–50], whereas five studies used reminder texts [41, 42, 44, 46, 47], four studies included feedback [43, 45–47], three studies used phone calls [38, 44, 49] and two studies used email messages [44, 46], as intervention components.

Almost all studies’ interventions were successful in achieving their primary objectives; however, three studies indicated that they were unsuccessful in achieving their secondary objectives [42, 45, 49]. Eleven studies outlined the conditions that influence the effectiveness of the intervention [14, 37, 39, 40, 42, 43, 46–50]. Out of the 15 included studies, only 2 clearly articulated their programme theory. Social cognitive theory and social–ecological model were the programme theories that were reported in these two studies [42, 46] (Supporting Information File 2).

### 3.3 Intervention Components and How the Intervention Was Intended to Work

Overall, intervention components were similar across studies, despite variations in the combination of intervention components. Education in the form of (seminars, lectures, presentations, interactions and giving reading materials), reminders, feedback, one‐on‐one counselling, environmental modifications, providing social support and follow‐up were the most frequently incorporated intervention components.

As we explained before, except for two studies [42, 46], other studies did not specifically state the programme theory or how they intended to operate. Using the retrieved results (Supporting Information File 2), we then attempted to explain how each intervention's components link to the theory for the remaining studies (Table 2). This makes it easier for us to explain and condense how the programme theory works.

All 15 included studies employed education as a component of the intervention in the form of lectures [47, 49, 50], training [44], presentation [38, 41], counselling [14, 40, 48, 50], seminars [14, 43, 45], phone‐ or internet‐assisted learning [42, 46], giving reading materials [39, 41, 43, 48, 50] and using posters [37, 39, 47]. These goals were to raise awareness of healthy lifestyles, improve knowledge, skills, attitudes and practise and enhance the components of behaviours, like self‐efficacy, intention and subjective norms towards a healthy lifestyle.

Reminders were utilized as intervention components in seven studies in the form of text messages, phone calls and emails [38, 41, 42, 44, 46, 47, 49]. These were meant to serve as a reminder of the essential elements of behaviours that had been emphasized throughout training, as well as to encourage adherence to the intervention, inspire them to do physical activity and promote healthy eating. It served as a follow‐up measure as well. In one study [38], the participants received a call from the researcher and were provided with a contact number for any inquiries. This approach aimed to promote interpersonal connections and interactions, fostering a sense of confidence, security and motivation to enhance their behaviour.

Four studies provided participants with feedback on their intervention adherence to motivate them [43, 45–47].

One‐on‐one counselling was employed to give counselling that was precisely tailored to the needs of the participants [40]. Every 6 months, a follow‐up counselling session was used as an intervention component to monitor participant progress, alert at‐risk participants and follow up with in‐person lifestyle counselling or a referral for medical care as needed [14].

In one study [42], health counsellors established agreements with participants and set attainable goals. Assignments combining educational material with positive reinforcement and behavioural modification strategies were given. Books were also given as incentives, which taught them to reward themselves by making time to relax. Respected community members served as role models. The objectives were to improve self‐efficacy, reinforce behaviour change and provide opportunities for observational learning. To improve their degree of involvement, self‐monitoring techniques like weighing in and submitting diet records and physical activity logs for each session were also used [43].

**FIGURE 2:**
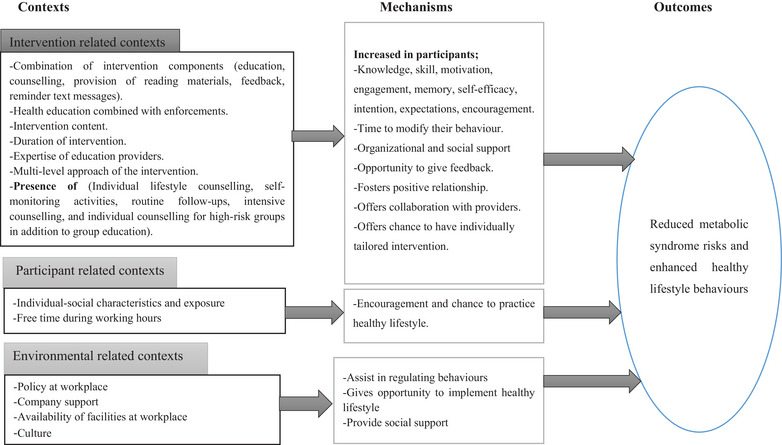
Context–mechanism–outcomes configuration of workplace‐based lifestyle education interventions summarized from the reviews.

In another study [44], there were pedometers available, and suggestions based on needs were also given. The goals were to support physical exercise and encourage adherence. In one study [14], there were opportunities to access medical treatment as needed, and more counselling was also available following the session. These triggered the participants to follow the suggested interventions. Group interaction activities, such as creating food pyramids and quizzing participants on the amount of sugar in various drinks, were used. This encourages interaction and enhances the ability to recognize a healthy diet [45]. These contextual factors provide the opportunity to act. Participants in a study [46] were asked to submit their self‐reported weights via a simple link to the software, encouraging actions. Smoking bans in the workplace [37, 47], changes to food menus [39], including head managers, family members and food handlers in the intervention [50], as well as showing a real case as an example [41], were implemented in those studies. These initiatives encourage leading healthy lifestyles, create opportunities to receive organizational and social support and provide learning opportunities by observing (Figure 2).

### 3.4 Mechanisms of Action

The combination of intervention components, including education, counselling and the provision of reading materials, reminders and feedback, contributes to the effectiveness of healthy lifestyle education interventions by increasing the knowledge, memory and skills (behavioural capability) for leading a healthy lifestyle, intention, motivation, self‐efficacy and expectations of the participants [42].

Health education combined with enforcement (such as smoking bans) at the workplace can contribute to the success of healthy lifestyle interventions by increasing the awareness of the seriousness of diseases (such as metabolic syndrome) and the significance of adhering to intervention instructions to control the diseases. Enforcement of smoking bans also encourages users to quit and reinforces the workplace focus on health [65, 66].

Routine follow‐up increases the efficacy of interventions by allowing for the clarification of misunderstandings, the possibility of additional assessments and the adjustment of intervention components [67].

Self‐monitoring activities (e.g. self‐weighing, keeping track of how many cigarettes they smoke each day and keeping track of their diet) increase self‐awareness of target behaviours and outcomes, which can act as an early warning system if problems are developing and can help track the success [68]. It also helps participants identify their strengths and weaknesses, set realistic goals, track their progress and the tracking of their progress sustaining motivations.

Individual counselling for high‐risk individuals in addition to group education can increase links with providers, and engagements and give a more personal atmosphere where they can feel comfortable discussing difficult themes. This will improve intervention adherence and outcomes. It also offers the chance to create a more individualized intervention strategy that is tailored to the needs of the client. The interventionist can also give feedback and keep tabs on the person's development. Additionally, it fosters positive relationships and contributes to the participants’ success and adherence to the intervention [69].

Interventions that cover a wide range of contents (nutrition, physical exercise and psychological aspects), conducted for a long term, and have an expert to conduct the intervention allow the participants to access detailed information, get enough time to modify their behaviours, good knowledge and obtain the ability to develop a healthy lifestyle [42, 43].

The multilevel approach or inclusion of head managers and family members in the intervention helps the managers comprehend the intervention's health benefits. This prompts them to assist their staff by permitting them to attend follow‐up appointments and engage in physical activities [70]. As a result, the workers have more free time to adopt a healthy lifestyle. Family members who take part in the intervention provide social support [71].

The participants’ social characteristics and their exposure to the intervention or follow‐ups drive them to engage in healthy behaviours [42, 72, 73]. The availability of free time during working hours and allowing employees to have dedicated free time for exercise during working hours promote the integration of physical activity into their daily routines [74].

Policies at the workplace, such as smoking prohibitions, help employees control their behaviour and persuade them to quit smoking [75]. The availability of facilities for physical activity, and options for accessing a healthy diet at the workplace, give opportunities for practicing healthy lifestyle behaviours [76]. The organizational support can contribute to the effectiveness of the intervention by providing facilities for physical activity and a healthy diet, as well as by setting workplace rules that forbid unhealthy lifestyle practices like smoking [65]. This prevents organization‐related impediments or indirectly encourages employees to adhere to the intervention. The country or area culture that discourages unhealthy lifestyle choices, like giving tobacco as a gift, encourages people to follow intervention recommendations, even in the workplace [64].

In each of the 15 studies, participants interact with the intervention components by applying their reasoning. Their ability to respond to the resources (intervention components) is based on their drive and empowerment. This involvement brings about change, which then enhances the primary and/or secondary process outcomes of the study (Supporting Information File 2).

### 3.5 Study Outcomes

The majority of the studies were successful in achieving their primary objectives, such as managing metabolic syndrome and its components, as well as improving healthy lifestyle behaviours. However, in a study conducted among factory workers in Iran, the intervention group displayed higher levels of postprandial plasma glucose (a test given 2 h after starting a meal), LDL cholesterol, mean values for diastolic BP and fasting blood sugar compared to the control group [39]. Additionally, another investigation carried out in Iran did not find any significant changes in the intervention group for the secondary outcomes of waist circumference and BP [42]. Similarly, a study conducted in Malaysia did not find any notable differences between the intervention and control groups in terms of self‐perceived physical activity levels or exercise behaviours [45]. Furthermore, another Malaysian study reported an increase in hip circumference in the intervention group compared to the control group but did not observe any discernible change in steps per day or calorie intake [49] (Supporting Information File 2).

The low‐intensity level of health education programmes (programmes that only provide medical assessment, behavioural counselling, web‐based health programmes and/or a less frequent follow‐up are considered low intensity) (14), a passive intervention (an intervention that depends only on the volunteer of the participants), short intervention period (49), cultural practices such as giving tobacco as gifts (39), the tight work schedule of the participants, or time‐poor (50, 52), lack of closer monitoring and supervision, short‐time meeting throughout the study period, lack of either an individually prescribed diet or a personal dietary counselling approach and lack of the individual level and tailored physical activity intervention design to each individual's baseline condition (51), were identified as hindering the effectiveness of the interventions in the reviewed studies.

## 4 Discussion

This review confirms the importance of considering intervention‐related contextual factors in intervention design, such as combining components (e.g. adding reminders and feedback to in‐person education as intervention components), enforcing health regulations (e.g. smoking bans), incorporating follow‐up and self‐monitoring activities, employing multilevel approaches and providing individual counselling alongside group education. These intervention‐related factors significantly impact motivation, compliance and other components of behaviours to bring positive outcomes in managing metabolic syndrome and behaviour modification.

Other studies support these findings, highlighting the benefits of tailored feedback, reminders, smoking bans, regular follow‐up, self‐monitoring activities and individualized counselling. A study from The Netherlands [57] suggests that tailored feedback and email reminders increase intervention appeal and adherence. Another review reveals that workplace smoking bans lead to reduced smoking rates and increased quitting intentions [75]. Regular follow‐up improves motivation and compliance, as shown in a study [77]. Including self‐monitoring activities in interventions improves behaviour changes and intervention outcomes, as supported by a systematic review [78]. Research from Japan demonstrates that individual counselling reduces multiple metabolic syndrome markers, whereas group counselling reduces specific markers [79]. Another randomized controlled trial carried out in the town of Enschede in The Netherlands also revealed that individual face‐to‐face counselling has a positive impact on multiple health indicators. The study demonstrated that individuals who received individual face‐to‐face counselling on physical activity, fitness and health in the workplace experienced significant improvements in blood cholesterol levels, body fat percentage and overall energy expenditure [62]. An umbrella review was done on key factors associated with adherence to physical exercise in patients with chronic diseases and older adults, which also verified that adherence to physical activity is impacted by the involvement of experts from different fields, social support and relatedness [63].

Our review further confirms that the effectiveness of the intervention is influenced by participant‐related contextual factors, including individual social characteristics and the availability of free time during working hours. Participants’ social characteristics, such as their social support networks, social norms and interpersonal relationships, can influence their engagement and adherence to interventions. For example, individuals with strong social support systems may be more motivated and encouraged to participate actively in the intervention. On the other hand, individuals facing social barriers or lacking support may require additional strategies to overcome these challenges and maintain their participation. When employees have dedicated time for exercise or other health‐promoting activities within their work schedule, it becomes more convenient and feasible for them to engage in such behaviours. This can lead to increased adherence and sustained participation in the intervention. This finding aligns with an umbrella review that investigated factors influencing adherence to physical exercise in patients with chronic diseases and older adults. The review confirmed that participant characteristics, social support and relatedness significantly impact adherence to physical activity [63].

This review also emphasizes the importance of considering environmental contextual factors when designing education interventions for managing metabolic syndrome and promoting healthy behaviours. These factors include workplace policy, workplace support, the availability of facilities at the workplace and the local culture. Taking these environmental contexts into account enhances the effectiveness and relevance of the interventions. Policies promoting smoke‐free environments, healthy food options and opportunities for physical activity during work hours create a supportive environment for healthier choices. When the workplace demonstrates a commitment to employee health through resources and encouragement, it fosters a culture that motivates healthy behaviours. Workplace facilities like fitness centres, walking paths and healthy food options facilitate employee engagement in physical activity and healthy eating. Tailoring interventions to align with cultural norms and addressing cultural barriers enhances resonance and effectiveness among the target population. The findings from a review study on enhancing workplace health and well‐being through cultural reform support the notion that managerial and organizational support can mediate the effects of workplace interventions [80].

Overall, this realist review has identified contextual factors, including strong social support systems, workplace influence, involvement of worksite managers and cultural relevance that contribute to the effectiveness of workplace‐based education interventions for managing metabolic syndrome in LMICs, which may not be as prominent in developed countries. In LMICs, social support systems, like those within families and the community, are often strong; workplaces serve as major social interaction and support centres for people; workplace managers have a greater direct and significant influence over workers; and cultural sensitivity is also high [81, 82].

This realist review has strengths in addressing policy‐relevant questions regarding the effectiveness of workplace interventions for controlling metabolic syndrome. It also identified specific contextual factors that affect the efficacy of these interventions in LMICs. However, this review does have certain limitations. First, the review focuses only on contextual factors related to the intervention, participants and environment, potentially overlooking other influential factors. Second, because only 2 of the 15 studies specifically mentioned their programme theories, we might not be able to match the specific programme theories with intervention explanations for other included studies. Third, the exclusion of non‐English publications may have impacted the scope of the findings.

The findings of this review have important implications for policymakers and researchers. While designing workplace‐based education interventions for managing metabolic syndrome and promoting healthy lifestyle behaviours in LMICs, several key considerations should be taken into account. These include carefully selecting effective intervention components, incorporating follow‐ups and individualized approaches, engaging workplace leaders and family members, adapting the intervention to local culture and participants’ contexts and creating a supportive work environment. Additionally, it is critical to take internet and mobile network accessibility into account when designing interventions to improve communication and information sharing in these settings.

## 5 Conclusions

The review concluded that while developing and putting into practice healthy lifestyle policies in the workplace, policymakers and researchers should take social support, workplace influences, manager involvement and cultural relevance into account. Taking these contextual aspects into consideration will contribute to the efficacy and long‐term viability of these interventions, especially in settings with lower and middle‐income.

Our model for comprehending interventions in healthy lifestyle education also shows how context influences the mechanisms that affect participants' responses to the intervention.

## Author Contributions

S.K.B. and M.K. conceived the study. S.K.B. and T.T.A. searched, extracted data and assessed the quality. S.K.B. analysed, interpreted the result and developed the manuscript. M.K. and H.S. critically reviewed the manuscript for intellectual content. All authors read and approved this manuscript. S.K.B. is the guarantor for the study.

## Ethics Statement

The authors have nothing to report.

## Conflicts of Interest

The authors declare no conflicts of interest.

## Supporting information

Supporting Information

Supporting Information

Supporting Information

Supporting Information

Supporting Information

## Data Availability

The data that support the findings of this study are included in the submission document.
