# Supplementary material for: Workplace‐Based Education Interventions for Managing Metabolic Syndrome in Low‐ and Middle‐Income Countries: A Realist Review
Source: Public Health Chall. 2024 Jul 23;3(3):e224. doi: 10.1002/puh2.224 (PMC12039584; doi:10.1002/puh2.224)
Supplement: Supplementary file 1 — Supporting Information [file PUH2-3-e224-s002.docx]

**Database search strategies and results of a workplace-based education intervention for managing metabolic syndrome in low- and middle-income countries: a realist review**

1. **PubMed search techniques and results;**

| **Serial**  **number** | **Themes** | **Search terms and combinations** | **Results** |
| --- | --- | --- | --- |
| 1 | Metabolic syndrome-related | "Metabolic Syndrome" OR "MetS" OR "Dysmetabolic Syndrome" OR "Cardio metabolic Syndrome" OR "Cardio metabolic risk factors" OR "Metabolic X Syndrome" OR "Syndrome X" OR "deadly quartet" OR "insulin resistance syndrome" OR "Reaven's Syndrome" OR "metabolic risk factors" OR dyslipidemia* OR dyslipidaemia* OR Hyperlipidemia* OR hyperlipidaemia OR "high blood cholesterol" OR hypercholesterolemia OR hypercholesterolaemia OR triglycerides OR hypertriglyceridemia OR hypertriglyceridemia OR biomarkers OR overweight OR obesity OR obese OR "body mass index" OR "waist circumference" OR "waist to hip ratio" OR "weight reduction" OR "weight management" OR "blood glucose" OR impaired OR "glucose tolerance" OR "glucose intolerance" OR "high blood glucose" OR hyperglycemia OR hyperglycaemia OR "High fasting" OR "high fasting blood glucose" OR "plasma glucose" OR "insulin resistance" OR diabetes OR "diabetes mellitus" OR "blood pressure" OR "high blood pressure" OR hypertension OR "systolic blood pressure" OR "diastolic blood pressure" | 3,970,179 |
| 2 | Education related | education OR "health education" OR "health education intervention" OR prevention OR "lifestyle education" OR teach* OR training OR "health promotion" OR "lifestyle and health promotion" OR "psychological and motivational interventions" OR "health literacy" OR advice OR counselling OR "healthy lifestyle education" OR "metabolic risk reduction education" OR "sedentary behaviour reduction education" | 5,433,839 |
| 3 | Employed adults related | employed OR “employed adults” OR "working adults" OR "employed people" OR office OR "office workers" OR "office jobs" OR "government office worker" OR "government workers" OR "government employees" OR "occupational groups" OR "government staff" OR "government office employees" | 1,025,129 |
| 4 | Combination of  themes | #1 AND #2 AND #3 | 35,463 |
| 5 | Combination of  Themes and filters | #1 AND #2 AND #3 and after filtered by: Full text, Clinical Trial, Randomized Controlled Trial, Humans, English, Adult: 19+ years | 1,985 |

1. **Web of science search techniques and results**

| **Serial**  **number** | **Themes** | **Search terms and combinations** | **Results** |
| --- | --- | --- | --- |
| 1 | Metabolic syndrome related | TS=(“Metabolic Syndrome” OR “MetS” OR “Dysmetabolic Syndrome” OR “Cardio metabolic Syndrome” OR “Cardio metabolic risk factors” OR “Metabolic X Syndrome” OR “Syndrome X” OR “deadly quartet” OR “insulin resistance syndrome” OR “Reaven's Syndrome” OR “metabolic risk factors” OR dyslipidemia* OR dyslipidaemia* OR Hyperlipidemia* OR hyperlipidaemia OR “high blood cholesterol” OR hypercholesterolemia OR hypercholesterolaemia OR triglycerides OR hypertriglyceridemia OR hypertriglyceridemia OR biomarkers OR overweight OR obesity OR obese OR “body mass index” OR “waist circumference” OR “waist to hip ratio” OR “weight reduction” OR “weight management” OR “blood glucose” OR impaired OR “glucose tolerance” OR “glucose intolerance” OR “high blood glucose” OR hyperglycemia OR hyperglycaemia OR “High fasting” OR “high fasting blood glucose” OR “plasma glucose” OR “insulin resistance” OR diabetes OR “diabetes mellitus” OR “blood pressure” OR “high blood pressure” OR hypertension OR “systolic blood pressure” OR “diastolic blood pressure”) | 2,772,186 |
| 2 | Education related | TS=(education OR “health education” OR “health education intervention” OR prevention OR “lifestyle education” OR teach* OR training OR “health promotion” OR “lifestyle and health promotion” OR “psychological and motivational interventions” OR “health literacy” OR advice OR counselling OR “healthy lifestyle education” OR “metabolic risk reduction education” OR “sedentary behaviour reduction education” ) | 3,673,966 |
| 3 | Employed adults related | TS=(employed OR “employed adults” OR “working adults” OR “employed people” OR office OR “office workers” OR “office jobs” OR “government office worker” OR “government workers” OR “government employees” OR “occupational groups” OR “government staff” OR “government office employees” ) | 1,797,996 |
| 4 | Combination of  themes | #1 AND #2 AND #3 | 8,170 |
| 5 | Combination of  Themes and filters | #1 AND #2 AND #3 and after filtered by: document type (article), language ( English), topics (nutrition and diabetics, diabetics, nursing, health care policy), and web of science categories ( public environmental occupational health, medicine general internal, endocrinology metabolism, nutrition diabetics, health care sciences services) | 1,587 |

1. **ProQuest search techniques and results**

| **Serial**  **number** | **Themes** | **Search terms and combinations** | **Results** |
| --- | --- | --- | --- |
| 1 | Metabolic syndrome related | Title=(“Metabolic Syndrome” OR “MetS” OR “Dysmetabolic Syndrome” OR “Cardio metabolic Syndrome” OR “Cardio metabolic risk factors” OR “Metabolic X Syndrome” OR “Syndrome X” OR “deadly quartet” OR “insulin resistance syndrome” OR “Reaven's Syndrome” OR “metabolic risk factors” OR dyslipidemia* OR dyslipidaemia* OR Hyperlipidemia* OR hyperlipidaemia OR “high blood cholesterol” OR hypercholesterolemia OR hypercholesterolaemia OR triglycerides OR hypertriglyceridemia OR hypertriglyceridemia OR biomarkers OR overweight OR obesity OR obese OR “body mass index” OR “waist circumference” OR “waist to hip ratio” OR “weight reduction” OR “weight management” OR “blood glucose” OR impaired OR “glucose tolerance” OR “glucose intolerance” OR “high blood glucose” OR hyperglycemia OR hyperglycaemia OR “High fasting” OR “high fasting blood glucose” OR “plasma glucose” OR “insulin resistance” OR diabetes OR “diabetes mellitus” OR “blood pressure” OR “high blood pressure” OR hypertension OR “systolic blood pressure” OR “diastolic blood pressure” ) | 1,292,332 |
| 2 | Education related | Title= (education OR “health education” OR “health education intervention” OR prevention OR “lifestyle education” OR teach* OR training OR “health promotion” OR “lifestyle and health promotion” OR “psychological and motivational interventions” OR “health literacy” OR advice OR counselling OR “healthy lifestyle education” OR “metabolic risk reduction education” OR “sedentary behaviour reduction education” ) | 7,746,871 |
| 3 | Employed adults related | Title=(employed OR “employed adults” OR “working adults” OR “employed people” OR office OR “office workers” OR “office jobs” OR “government office worker” OR “government workers” OR “government employees” OR “occupational groups” OR “government staff” OR “government office employees” ) | 5,706,381 |
| 4 | Combination of  themes | #1 AND #2 AND #3 | 272 |
| 5 | Combination of  Themes and filters | #1 AND #2 AND #3 and after filtered by: Language (English) | 271 |

1. **Scopus search techniques and results**

| **Serial**  **number** | **Themes** | **Search terms and combinations** | **Results** |
| --- | --- | --- | --- |
| 1 | Metabolic syndrome related | TITLE-ABS-KEY ("Metabolic Syndrome" OR "MetS" OR "Dysmetabolic Syndrome" OR "Cardio metabolic Syndrome" OR "Cardio metabolic risk factors" OR "Metabolic X Syndrome" OR "Syndrome X" OR "deadly quartet" OR "insulin resistance syndrome" OR "Reaven's Syndrome" OR "metabolic risk factors" OR dyslipidemia* OR dyslipidaemia* OR hyperlipidemia* OR hyperlipidaemia OR "high blood cholesterol" OR hypercholesterolemia OR hypercholesterolaemia OR triglycerides OR hypertriglyceridemia OR hypertriglyceridemia OR biomarkers OR overweight OR obesity OR obese OR "body mass index" OR "waist circumference" OR "waist to hip ratio" OR "weight reduction" OR "weight management" OR "blood glucose" OR impaired OR "glucose tolerance" OR "glucose intolerance" OR "high blood glucose" OR hyperglycemia OR hyperglycaemia OR "High fasting" OR "high fasting blood glucose" OR "plasma glucose" OR "insulin resistance" OR diabetes OR "diabetes mellitus" OR "blood pressure" OR "high blood pressure" OR hypertension OR "systolic blood pressure" OR "diastolic blood pressure" ) | 3,918,675 |
| 2 | Education related | TITLE-ABS-KEY (education OR "health education" OR "health education intervention" OR “prevention” OR "lifestyle education" OR teach* OR training OR "health promotion" OR "lifestyle and health promotion" OR "psychological and motivational interventions" OR "health literacy" OR advice OR counselling OR "healthy lifestyle education" OR "metabolic risk reduction education" OR "sedentary behaviour reduction education" ) | 5,561,810 |
| 3 | Employed adults related | TITLE-ABS-KEY ( employed OR “employed adult” OR "working adults" OR "employed people" OR office OR "office workers" OR "office jobs" OR "government office worker" OR "government workers" OR "government employees" OR "occupational groups" OR "government staff" OR "government office employees" ) | 1,738,576 |
| 4 | Combination of  themes | #1 AND #2 AND #3 | 9,264 |
| 5 | Combination of  Themes and filters | #1 AND #2 AND #3 and after filtered by: subject area (medicine, biochemistry, nursing, health professions), language (English), document type (Article), keywords (human, humans, article)  **NB.** Developed Countries such as the US, Canada, Australia, Italy, Poland, France, Germany, Japan, Spain, the United Kingdom, Norway, Austria, Portugal, Belgium, Netherlands, and Sweden were excluded from the search. | 1,631 |

1. **PsycINFO search techniques and results**

| **Serial**  **number** | **Themes** | **Search terms and combinations** | **Results** |
| --- | --- | --- | --- |
| 1 | Metabolic syndrome-related | TITLE-ABS-KEY ("Metabolic Syndrome" OR "MetS" OR "Dysmetabolic Syndrome" OR "Cardio metabolic Syndrome" OR "Cardio metabolic risk factors" OR "Metabolic X Syndrome" OR "Syndrome X" OR "deadly quartet" OR "insulin resistance syndrome" OR "Reaven's Syndrome" OR "metabolic risk factors" OR dyslipidemia* OR dyslipidaemia* OR hyperlipidemia* OR hyperlipidaemia OR "high blood cholesterol" OR hypercholesterolemia OR hypercholesterolaemia OR triglycerides OR hypertriglyceridemia OR hypertriglyceridemia OR biomarkers OR overweight OR obesity OR obese OR "body mass index" OR "waist circumference" OR "waist to hip ratio" OR "weight reduction" OR "weight management" OR "blood glucose" OR impaired OR "glucose tolerance" OR "glucose intolerance" OR "high blood glucose" OR hyperglycemia OR hyperglycaemia OR "High fasting" OR "high fasting blood glucose" OR "plasma glucose" OR "insulin resistance" OR diabetes OR "diabetes mellitus" OR "blood pressure" OR "high blood pressure" OR hypertension OR "systolic blood pressure" OR "diastolic blood pressure" ) | 246,063 |
| 2 | Education related | TITLE-ABS-KEY (education OR "health education" OR "health education intervention" OR “prevention” OR "lifestyle education" OR teach* OR training OR "health promotion" OR "lifestyle and health promotion" OR "psychological and motivational interventions" OR "health literacy" OR advice OR counselling OR "healthy lifestyle education" OR "metabolic risk reduction education" OR "sedentary behaviour reduction education" ) | 974393 |
| 3 | Employed adults related | TITLE-ABS-KEY (employed OR “employed adults” OR "working adults” OR “employed people” OR office OR “office workers” OR “office jobs” OR “government office worker” OR “government workers” OR “government employees” OR “occupational groups” OR “government staff” OR “government office employees”) | 133,933 |
| 4 | Combination of  themes | #1 AND #2 AND #3 and after filtered by: full text, abstract, latest update, impact statement, APA psycArticles, Humans, Test DOI, All journals, English language, Open Access | 1404 |
