# Supplementary material for: Workplace‐Based Education Interventions for Managing Metabolic Syndrome in Low‐ and Middle‐Income Countries: A Realist Review
Source: Public Health Chall. 2024 Jul 23;3(3):e224. doi: 10.1002/puh2.224 (PMC12039584; doi:10.1002/puh2.224)
Supplement: Supplementary file 2 — Supporting Information [file PUH2-3-e224-s003.docx]

**Data extraction table of Workplace-based education interventions for managing metabolic syndrome in low- and middle-income countries: a realist review**

| **Author and year** | **Country** | **Research design, participant sample, setting, main outcome measure, and secondary outcome** | **Intervention description, intervention components, study duration, intervention providers** | **Intervention effect on primary, secondary, and process outcomes and overview** | **Authors’ program theory for the intervention (if stated) or probable program theory, based on the description of interventions (including likely mechanism/intervention function)** | | **Did the intervention work as intended or not?** | |
| --- | --- | --- | --- | --- | --- | --- | --- | --- |
| Jalal Abdi et al, 2015 | Iran | **Design:** RCT **Sample:** Employees with BMI >25kg/m2, working for at least 8 hours per week, having a computer and are familiar with working with a computer, access to the telephone, cell phone, and the Internet, and familiarity with working with these technologies, age above 18 years. n=435 total. **Setting:** Government offices **Main outcome measure:** weight, and changes in the constructs of the social cognitive theory 6 and 9 months after the intervention. **Secondary outcome:** Blood pressure and waist circumference | **Intervention:** Web and phone-assisted education intervention **Web-assisted intervention:** A website called “healthy employee “was designed for the intervention. A unique username and password were assigned for each participant and intervention started when the employees received a welcome e-mail. The employee was asked to fill out some personal details for the counselor. The employee then started with the first module.  **Intervention components:**  -Information about healthy nutrition and physical activity skills -Brochures **Phone intervention:** A cell phone line was developed for the intervention. The intervention started when the counselor first called the participant about two weeks after randomization. In this first contact the counsellor explained the goals of the” life style program”. SMS messages were sent every two weeks. I**ntervention-components:** -Modules about diet and physical activity Assignments -SMS messages -Brochures **Study-duration:**6months **Intervention provider:** Health advisors | **primary outcomes:** Weight loss: Telephone-assisted lifestyle intervention: 1.92 Kg  Web-assisted lifestyle intervention: 1.08 kg **Scores of social cognitive theory construct:** In the section of physical activity in the telephone-assisted group: The mean scores of self-efficacies increased from 22.73 (±4.47) to 26.3 (±3.97). In the section on physical activity in the web-assisted group: The mean scores of outcome expectancies increased from 22.70 (±2.61) to 26.36 (±2.14) during the follow-up period and 21.69 (2.29) during the maintenance phases, respectively. The mean scores of nutrition self-efficacy in the telephone-assisted group increased from 28.18 (±4.74) to 35.29 (±4.46) at the follow-up period and 30.17 (4.70) at the maintenance phase, respectively. **Secondary outcome:** Waist circumference and blood pressure: did not result in  significant changes.  **Process outcomes:** Between baseline and nine months after intervention up to 34 subjects withdrew from the study. Most employees withdrew because of personal reasons and lack of time. Two participants withdrew due to musculoskeletal disorders and one withdrew because of pregnancy. **Outcome overview:** The lifestyle intervention resulted in a weight loss of 1.92 and 1.08 kg in the telephone-assisted and web-assisted intervention groups, respectively. | Social-cognitive theory | | **Yes.** There is weight loss in the telephone-assisted and web-assisted intervention groups and increased mean scores of the constructs of self-efficacy, environment, outcome expectations, and outcome expectancies.  **No.** Waist circumference and Blood pressure did not result in significant changes. | |
| Siti Noraida Jamal et.al, 2016 | Malaysia | **Design:** RCT **Sample:** Employees of a public university in Kuala Lumpur, Bahasa Malaysia (national language of the country) literate, with a BMI of 27.5kg/m2 or more, and able to walk briskly for at least 10 minutes without assistance. n=194 total **Setting:** University **Main outcome measure:** Body mass index, Waist and hip circumference, Blood pressure, fasting blood glucose, fasting serum triglyceride, fasting serum cholesterol, HDL, LDL **Secondary outcome:** Physical activity, psychological (eating self-efficacy, social support, and negative thoughts) and quality of life. | **Intervention:** Group Support Lifestyle Modification. It was delivered through three seminars and five 90-minute sessions in two phases. The core phase (Phase 1) was run once in two weeks for three months, beginning with a two-day seminar. A fade frequency (Phase 2) started with a one-day seminar, followed by two 90-minute sessions, and concluded with a half-day seminar at the end of the active intervention. The first 30 to 40 minutes were on knowledge transfer while the subsequent duration was on practical /hands on experience such as food portion, food tasting, food weighing, aerobic exercise as well as cognitive restructuring. **Intervention components:** Seminars, providing reading materials, log book for diet and physical activity records, Self-monitoring activities including weighing-in and providing feedback. **Study duration:** 36 Weeks **Intervention provider:** Experts in diet/nutrition, sports medicine, and cognitive behavior psychology. | **primary outcomes:** Weight loss: 19 (19.6%) achieved 6% targeted weight loss.  **Secondary outcome:** Improved self-efficacy in dietary control, achieved better friend support and quality of life.  **Process outcome:** Attendance**:** Of the total six sessions within the first 12 weeks, 33 (17.0%) participants in the intervention group attended more than four sessions, 34 (17.5%) attended three to four sessions while 30 (15.5%) attended two sessions and less. During the fade frequency with four sessions, 20.6 percent of participants attended three to four sessions. Logbook submission: The overall logbook submission was unsatisfactory for both groups. Only 46.4% (n = 45) of intervention participants submitted their log books in the 12th week and 16.5% (n = 16) in the 24th week. Only 23.7% (23) comparison group participants submitted their log books at 12 weeks and 19.6% (19) at 24th week. **Outcome overview:** A total of 19.6% of the participants in the group support lifestyle modification achieved 6% weight loss compared to 4.1% in the comparison group (Risk Ratio 4.75; 95% CI: 1.68, 13.45). At 24 weeks, the retention rate was 83.5% for a group supporting lifestyle modification and 82.5% for comparison group. Group support lifestyle modification participants also achieved significant improvement in total weight self-efficacy score, negative emotions and physical discomfort subscales, Multidimensional Perceived Social Support friend subscale, and all domains in quality of life. Participants in the comparison group experienced a reduction in negative self-thoughts. | The programme theory was not reported in the study but the following characteristics were included.  Seminar with a focus on group learning, providing handouts.  Self-monitoring activities included weighing-in, submission of diet records, and physical activity log was also conducted for each session. Feedback was provided to motivate the participants in each session. | | **Yes.** There is weight loss, improved self-efficacy in dietary control, achieved better friend support and quality of life in the intervention group. |  |
| Usha Shrivastava et al, 2017 | India | **Design:** RCT **Sample:** Employees of both the public and private, from the different sites across Delhi and the National Capital Region. Willing to participate, age group of 25–55 yrs., and having BMI ≥ 23 kg/m2. n = 310 total. **Setting:** Both the public and private work sites across Delhi and the National Capital Region. **Main outcome measure:**  Weight, BMI, blood pressure, waist-hip ratio, biceps, triceps, subscapular, and suprailiac skinfolds, fasting blood glucose (FBG) and serum lipids [total cholesterol (TC), serum triglyceride (TG), and high-density lipoprotein cholesterol (HDL-c)]. **Secondary outcome:** Behavioral risk factors (tobacco and alcohol consumption), dietary intake, physical activity | **Intervention:** A multicomponent intervention which was implemented for 6 months for active intervention sites. Participants in the intervention group received detailed sessions on the different topics related to healthy living, diet, and physical activity supported by use of pedometers. Two sessions on each topic were conducted in the intervention sites every 15 days for 45–60 minutes. Subsequently, in these sessions, reinforcement and need-based advice were provided. Stress management sessions were also provided to the employees to cope with the workplace and other kinds of stress. **Intervention components:** -Training, reinforcement, and need-based advice, providing pedometers, Text messages, E-mails, and repeated phone calls.  **Study duration:** 6 months **Intervention provider:** physicians, nutritionist, and physical trainer. | **Primary outcomes:** Intervention resulted with significant decrease in the mean values of weight (1.60 ± 2.76). BMI (0.55 ± 0.96), waist circumference (1.87 ± 3.49), hip circumference (1.23 ± 2.94), W-HR (0.007 ± 0.029), all the four skinfolds (12.51 ± 10.38) (biceps, triceps, subscapular, and suprailiac), FBG (2.89 ± 6.48), TG (11.07 ± 46.56), and increase in HDL-c (−2.20 ± 5.37) in the intervention group. More than 5% weight loss was observed in 12% of the individuals in the intervention group as compared to only 4% of the individuals in the control group. The sum of all the skinfold measurements in the intervention group decreased significantly more than the control group (12.51 ± 10.38 versus 3.50 ± 8.18) respectively. **Secondary outcome:** A significant reduction was observed in the sedentary lifestyle. More individuals converted from sedentary to more active lifestyles (67% to 55%) in the intervention group as compared to the control group (69% to 65%). Dietary behavior also improved in terms of decrease in total calorie intake and fat consumption. **Process outcome:** not reported **Outcome overview:** At the end of the intervention, the following significant changes were observed in the intervention group: a decrease in weight, BMI, waist circumference, serum triglycerides, and an increase in HDL-c. Weight loss of more than 5% was seen in 12% and 4% of individuals in the intervention and control groups, respectively. Most importantly, the sum of all the skinfold measurements (mm) in the intervention group decreased significantly more than the control group (12.51 ± 10.38 versus 3.50 ± 8.18, respectively). More individuals converted from sedentary to more active lifestyles (67% to 55%) in the intervention group as compared to the control group (69% to 65%). | The programme theory was not reported in the study but the following characteristics were included.  Face-to-face sessions, reinforcement, and need-based advice were provided to increase employee awareness and improvement in knowledge, attitude, and practices toward a healthy diet.  Physical activity training sessions were given to encourage them to continue physical activity supported by the use of pedometers.  Stress management sessions were also provided to the employees to cope with the workplace and other kinds of stress. | | **Yes.** There were significant decreases in the mean values of weight, BMI, waist circumference, hip circumference, W-HR, all four skinfolds (biceps, triceps, subscapular, and suprailiac), FBG, TG, and increase in HDL-c in the intervention group.  A significant reduction in the sedentary lifestyle and improved in terms of decrease in total calorie intake and fat consumption was also observed. | |
| Eng JY et al,2016, | Malaysia | **Design:** Quasi-experimental  **Sample:** Employees aged 35 years and above, with at least one follows up measurements and no change in antihypertensive medication during the study period**.** n=1,365 total **Setting:** University **Main outcome measure:** Blood pressure **Secondary outcome:** No secondary outcomes reported | **Intervention:** Health promotion program: It was aimed to promote employees' health and well-being by increasing health awareness and promoting healthy lifestyle practices. The program was of low intensity (programs that only provide medical assessment, behavioral counseling, web-based health programs and/or a less frequent follow-up are considered as low intensity), and the activities included annual health screening and physical examination, health educational seminars, and health exhibitions. Mainly focused on a healthy diet, physical activity, quitting smoking, and stress management.  **Intervention components:** health screening, physical examination, health educational seminars, and health exhibitions, face-to-face lifestyle counseling, referral for medical treatment, when necessary, followed up with subsequent counseling, health brochures  **Study duration:** 6 years  **Intervention providers:** Dietitians | **Primary outcomes:**  Blood pressure: SBP improved by 0.1 mmHg per year and DBP improved by 0.2 mmHg per year. There was a reduction of almost 2 mmHg per year among participants from both hypertension and at risk of hypertension subgroups.  **Secondary outcome:** not reported in the study  **Process outcome:** not reported in the study  **Outcome overview:** The hypertension subgroup showed the greatest improvement in SBP (-2.36 mmHg per year), There was also significant improvement in SBP among those at risk of hypertension (-0.75 mmHg per year). There was a significant reduction in DBP over the study period (-0.22 mmHg per year). The reduction in DBP among the hypertensive subgroup (-1.76 mmHg per year) was larger than those in the at-risk subgroup (-0.56 mmHg per year). | The programme theory was not reported in the study but the following characteristics were included. -Employees who were found to be at risk of obesity, hypertension, hypercholesterolemia, and diabetes were notified and followed up with face-to-face lifestyle counseling or referral for medical treatment when necessary.  -Followed up with subsequent counseling sessions every 6 months to review their progress. | | **Yes.** There was a significant reduction in SPB and DBP over the study period. | |
| Ayiesah Ramli et al, 2013 | Malaysia | **Design:** non-randomized pilot **Sample:** Employees who had a body mass index (BMI) of 25 kg/m2 or above, and who were between 18 and 60 years old. n=28 total.   **Setting:** Higher Education in Putrajaya  **Main outcome measure:** Anthropometry (body fat and body weight) and physical fitness (cardiovascular fitness, upper body strength and endurance, abdominal strength and endurance, and lower body flexibility).  **Secondary outcome:** Participants’ self-perceived levels of physical activity and behaviors toward physical activity. | **Intervention:** Obesity health program, which consisted of two weekly unsupervised exercise sessions and monthly dietary/health education sessions.  **Intervention components:** Group exercise, distribution of workout sheet, group seminars, feedback, and interactive  activities including quiz  **Study duration:** 6 months  **Intervention provider:** physiotherapist | **Primary outcomes:** Significant differences in body fat percentages (t = 47, z = –2.58), gross VO2max, t = 141, z = -2.46, partial curl up repetition (t = 192.5, z = –3.27), and sit and reach distance (t = 195.5, z = –2.78,). **Secondary outcome:** No significant effects were observed on the self-perceived level of physical activity (P = 0.145) or behavior toward exercise (P = 0.393).  **Process outcome:** not reported **Outcome overview:**  Significant differences were observed in body fat percentage (P = 0.010), gross maximum oxygen uptake (VO2max, P = 0.014), partial curl-up repetition (P = 0.001), and sit and reach distance (P = 0.005). However, no significant effects were observed on body mass (P = 0.193), self-perceived level of physical activity (P = 0.145), or behavior toward exercise (P = 0.393). | The programme theory was not reported in the study but the following characteristics were included. -Workout sheets, which involved at least 30 minutes of exercise, were distributed monthly. -A group seminar was conducted monthly to gather information on dietary input. -Suggestions were given and discussed concerning food servings, calorie counts, and the retrieval of nutritional information when consuming foods. -Interactive activities such as forming food pyramids and testing beverages for different sugar levels were carried out through quiz sessions. | | **Yes.** There are significant changes on body fat percentages (t = 47, z = –2.58), gross VO2max, t = 141, z = -2.46, partial curl up repetition (t = 192.5, z = –3.27), and sit and reach distance (t = 195.5, z = –2.78,).  **No.** no significant changes were observed in secondary outcomes. | |
| Patricia Constante Jaime et al, 2013, | Brazil | **Design:** A controlled community trial  **Sample:** workers aged 18–64 years with a BMI between 20.0 and 39.9 kg/m2. n=281. **Setting:** Pharmaceutical, communication, and service-sector companies.  **Main outcome measure:** weight, BMI, and waist circumference  **Secondary outcome:** Adherence | **Intervention:** Practical recommendations for diet and physical activity in the form of short standard texts. -Once registered to use the program, workers received a welcome message and practical recommendations for diet and physical activity in the form of short standard texts. -One month later, a new message was sent by email, reminding participants to report their self-measured weights in the program using a quick link to the software. The intervention was supported by interactive software developed specifically to aid the self-monitoring of weight, entitled the Healthy Weight Program (HWP). **Intervention components:** Practical recommendation in the form of standard texts, reminder emails, and feedback **Study duration:** 6 months **Intervention provider:** not reported | **Primary outcomes:** Reduction of body weight = -0.73 kg, BMI =-0.26kg/m2, and waist circumference =-0.99cm.  **Secondary outcome:** The majority of intervention group participants adhered to the proposed intervention.  **Process outcome:** not reported  **Outcome overview:** The intervention resulted in significant reductions in weight, BMI and waist circumference in the intervention group compared with the control group. The impact of the intervention-on-intervention group individuals’ body weight was 20.73 kg, while the weight of control group individuals increased. Intervention group individuals with adequate initial weights did not show significant variations, while those who were overweight demonstrated a significant reduction in body weight. The intervention resulted in a reduction of 0.26kg/m2 in BMI and 0.99cm in waist circumference, and the sustainability analysis after 12 months showed a continued reduction in body weight (20.72kg). | | Social cognitive theory to the proposed intervention. Model of planning of health promotion programs to design, implement, and assessment of intervention | **Yes.** There were significant reductions in weight, BMI, and waist circumference in the intervention group. | |
| Denis Anthony et al, 2015, | China, India, and Mexico | **Design:** Nonrandomized control trial  **Setting:** industrial, health and school workplace **Sample:** All workers aged 18–64 years in each workplace  **Main outcome measure:** Reduce tobacco use, increase physical activity, and improve dietary intake.  **Secondary outcome:** not reported | **Intervention:** Health education about hazards of tobacco and passive smoking, health benefits of physical activity, and healthy eating. structural change, and community mobilization policies, mandates, and restrictions included no smoking days and smoking bans in the workplace as well as incentives. **Intervention components:** Developed and displayed posters, organized work-break exercises, sports competitions, and mountain-climbing events, distributed vegetable seeds and fertilizer and provided training on vegetable cultivation, created marked walking paths around worksites, smoking bans in the workplace as well as incentives etc. **Study duration:** 18–24 months  **Intervention provider**: not reported | **Primary outcomes:** Tobacco use decreased in the intervention group by 6%, ≥5 portions of fruit and vegetables/day increased in the intervention and control group =6.9% vs. 1.5%. And only one third of the sample achieving recommended levels of activity.  **Secondary outcome:** not reported  **Process outcome:** not reported **Outcome overview:** The prevalence of tobacco use reduced significantly in men (6.0%, p < .001) and the proportion eating five portions of fruit and vegetables daily increased (+6.9%, p < .001) compared with the control group. There were no significant differences between the groups for changes in physical activity or prevalence of overweight. | The programme theory was not reported in the study but the following characteristics were included.  Developed and displayed posters about hazards of tobacco and passive smoking, health benefits of physical activity, and healthy eating.   Developed and implemented tobacco-free policy Developed and displayed No smoking signs Creation of special “tobacco police” who enforced smoke-free public spaces Displayed point of decision (POD) prompts by lifts and stairwells Organized work-break exercises, sports competitions, and mountain-climbing events Introduced healthy options to canteens Displayed POD prompts in canteens and restaurants | | **Yes.** There is significant reduction of tobacco use in men and increased of the proportion eating five portions of fruit and vegetables daily in intervention group compared with the control group. | |
| Zengwu Wang et al, 2020 | China | **Design:** A cluster randomized clinical trial  **Setting:** In 60 workplaces across 20 urban regions in China.  **Sample:** Employees with hypertension, aged 18 to 60 years, contracted employee, signed consent form, and agreed to participate for 2 years. n=4166 total.  **Main outcome measure:** Change in BP control rate  **Secondary outcome:** Changes in BP level and smoking, drinking, exercise, and overweight or obesity. | **Intervention:** A workplace wellness program for improving employees’ cardiovascular health which include CVD health education, a reasonable diet, tobacco cessation, physical environment promotion, physical activity, stress management, and health screening. And a programme for managing hypertension based on guidelines with a community health center intervention and monthly visits to achieve blood pressure control. **Intervention components:** lectures, posters, and text messages, nutrition education and/or healthy diet information to the employees, tobacco control regulation in the workplaces, modifying workstations and office layouts, accessible indoor or outdoor sports facilities, including an indoor walking path, meditation, tai chi, or deep breathing, annual health check-ups and feedback to identify key risk factors. **Study duration:** 24 months **Intervention provider:** not reported | **Primary outcomes:** BP control rate: intervention group= 66.2%, control group=44.0% and the overall intervention effect was higher (OR, 1.77; 95% CI, 1.58-2.00)**. Secondary outcome: -**SBP changed by −10.5 mm Hg (95% CI, -10.9 to -10.0 mm Hg) from baseline in the intervention group and by −4.7 mm Hg (95% CI, −5.5 to −3.9 mm Hg) in the control group. -DBP changes of −7.3 mm Hg (95% CI, −7.6 to −6.9 mm Hg) in the intervention group and −3.6 mm Hg (95% CI, −4.3 to −3.0 mm Hg) in the control group.  -Drinking (−18.4%; 95% CI, −20.6% to −16.2%), perceived stress (−22.9%; 95% CI, −24.8% to −21.1%), and excessive use of salt (−32.0%; 95% CI, −33.7% to −30.4%) as well as a substantial improvement in regular exercise (34.0%; 95% CI, 32.3%-35.6%) from baseline. Smoking (−8.4%; 95% CI, −10.7% to −6.1%), fatty food intake (−55.5%; 95% CI, −57.3% to −53.8%), and overweight or obesity (−6.7%; 95% CI, −8.4% to −5.0%).  **Process outcome:** Compliance with medication use increased during the follow-up period, reaching 93% at 24 months.  **Outcome overview:** The intervention group had significantly higher blood pressure control and a net reduction in systolic and diastolic blood pressure of 5.8- and 3.6-mm Hg compared with the control group, but the intervention effect on overweight or obesity was insignificant. | The programme theory was not reported in the study but the following characteristics were included.  -Providing nutrition education and/or healthy diet information to the employees and encouraged to select the healthy options when having lunch in the cafeteria. -Tobacco control regulation was developed to improve tobacco cessation rates. -Modifying workstations and office layouts to decrease sedentary behavior and increase movement.   -Encouraged participants to increase physical activity with exercise breaks during working hours.  -Accessible indoor or outdoor sports facilities, including an indoor walking path, were provided to engage employees in regular physical activity. -Relaxation techniques were provided monthly to help workers deal with stress. -The health screening component included annual health check-ups and feedback to identify key risk factors | | **Yes.** There is significantly higher blood pressure control and a net reduction in systolic and diastolic blood pressure and greater reduction was reported in the rates of drinking, perceived stress, and excessive use of salt. | |
| Maryam Nooritajer, 2010, | Iran | **Design:** Semi-experimental study  **Setting:** University **Sample:** Women who had at least one-year employment background and currently be an employee of Iran University of medical science and volunteer to participate. n=200 total.  **Main outcome measure:** Nutrition style and body mass index  **Secondary outcome:** not reported | **Intervention:** Education about nutrition style which was about using healthy diet and body weight control.  **Intervention components:** Face to face interview, booklet presentation, telephone call.  **Study duration:** 2 months **Intervention provider:** Researcher | **Primary outcomes:** Obesity and overweight decreased from 51.4% of women before the educational intervention to 48.5% after education intervention. Nutrition style improved in 72% of women after intervention.  **Secondary outcome:** not reported  **Process outcome:** not reported  **Outcome overview:** The changes of nutrition status after education had more effective on the women aged 35-44 years with 3.1±8.4 and the most changes were on BMI after education on nutrition style with 4.2±9 that had 2 children. Educational Measurement had improved nutrition style and reduced BMI in 72% of the employed women. The score of nutrition style were increased on comparative BMI (Mean and SD) before and after education (35.6 ± 8.9 vs. 39.2 ± 9.4, P<0.05). | The study did not report the programme theory, but it did include the following characteristics. Face-to-face interview about essential guidelines. The educational booklet was presented to the participants, and the researcher made a telephone call to them biweekly and also gave them her phone number so they could call her and ask any questions regarding educational booklet. | | **Yes.** Obesity and overweight decreased from 51.4% of women to 48.5% women and nutrition style improved in 72% of women. | |
| MARCOS AUSENKA RIBEIRO et al, 2014 | BRAZIL | **Design:** RCT **Setting:**  University hospital  **Sample:** Women age 40–50-year-old who were employees from a university hospital and physically inactive at their leisure time. n=195 total.  **Main outcome measure:** Total number of steps  **Secondary outcome:** weight and waist circumference | **Intervention:** 195 women were randomly assigned to one of four groups: minimal treatment comparator (MTC), pedometer-based individual counselling (PedIC), pedometer-based group counselling (PedGC), and aerobic training (AT).  - MTC were given general advice about the benefits of physical activity (PA), and received a booklet with information on how to increase PA in their daily lives. -PedIC received a similar intervention of the MTC and additionally received a pedometer. -PedGC received eight group counselling sessions, each lasting 60 min. The counselling goal aimed to achieve behavioral changes with the following approaches: identifying the benefits of improved PA, learning how to overcome the daily barriers to increase PA, self-monitoring PA using the pedometer, goal setting (an increase in the number of steps per day) and relapse prevention, and group walking (10 min of brisk walking and monitoring the number of steps). The target step number was the same as that used in the PedIC. -AT was received aerobic exercise training performed on a treadmill, and after a 5-min warm-up, the exercise intensity was based as moderate to intense followed by a 5-min cool down. **Intervention components:** General advice, counselling, booklet, dairy, pedometer  **Study duration:** 3 months  **Intervention provider:** Health professionals with experience in PA counselling, experienced exercise professional. | **Primary outcomes:** PedGC and PedIC significantly increased the total number of steps after 3 months to 1475 and 512 steps per day respectively, compared with the MTC (-597 steps per day, P < 0.05). Women from the AT group did not present any change in the total number of steps after 3 months (P ˃ 0.05).  **Secondary outcome:** A significant decrease in weight and waist circumference were observed in AT after 3 months by -0.700 (-1.193 to 0.986) and -0.95 (-2.20 to 0.29) respectively, when compared with PedIG and PedGC. **Process outcome:** Protocol adherence: 34 women dropped out; and the study adherence rate was approximately 80%. Lower participant adherence was observed in the AT (54.3%) and in the PedGC (70%) groups. In contrast, the MTC and the PedIC groups showed significant adherence (96% and 94.4%, respectively). **Outcome overview:** Individual (PedIC) and group counselling (PedGC) significantly increased the total number of steps after 3 months (512 and 1475 steps per day, respectively) compared with the MTC (-597 steps per day, P <0.05); however, the increase in the total number of steps observed in the PedGC group was even higher when compared with the PedIC group (P <0.05) and presented the larger Effect Size (1.4). In addition, an increase in the number of steps at moderate intensity was also observed in the PedGC group after 3 months when compared with the PedIC group and MTC (PedGC, 845 steps per day, P < 0.05). Women from the AT group did not present any change in the total number of steps after 3 months (P ˃ 0.05). A significant decrease in weight and waist circumference were observed after 3 months by -0.700 (-1.193 to 0.986) and -0.95 (-2.20 to 0.29) respectively when compared with PedIG and PedGC. The study adherence rate was approximately 80%. | The study did not report the programme theory, but it did include the following characteristics.  -The counselling was provided to achieve behavioral changes with the following approaches: identifying the benefits of improved PA, learning how to overcome the daily barriers to increase PA, self-monitoring PA using the pedometer, goal setting (an increase in the number of steps per day) and relapse prevention, and group walking (10 min of brisk walking and monitoring the number of steps). | | **Yes.** There are significant increases in total number of steps in PedGC and PedIC groups and significant decrease in weight and waist circumference were observed in AT after 3 months. | |
